# Differential bud activation by a net positive root signal explains branching phenotype in prostrate clonal herbs: a model

**DOI:** 10.1093/jxb/ert427

**Published:** 2014-01-07

**Authors:** R. G. Thomas, F. Y. Li, M. J. M. Hay

**Affiliations:** AgResearch Grasslands, Private Bag 11008, Palmerston North, New Zealand

**Keywords:** Axillary bud outgrowth, branching hierarchies, branching regulation, bud activation, bud outgrowth potential, predictive model, prostrate clonal herbs, root-derived signal, *Tradescantia fluminensis*, *Trifolium repens*.

## Abstract

Regulation of branching within perennial prostrate clonal herbs differs from the annual orthotropic species, *Arabidopsis* and pea, as the dominant signal transported from roots is a branching promoter, not an inhibitor. *Trifolium repens*, an exemplar of such prostrate species, was used to investigate the interaction between roots and branch development. This study tests whether or not current knowledge when synthesized into a predictive model is sufficient to simulate the branching pattern developing on the shoot distal to a basal root. The major concepts underpinning the model are: (i) bud outgrowth (activation) is stimulated in a dose-dependent manner by branching promoter signals from roots, (ii) the distribution of this net root stimulus (NRS) is uniform throughout the shoot system distal to the basal root but declines geometrically in intensity upon continued enlargement of this shoot system, and (iii) each bud has an outgrowth potential, equal to the activation level of the apical bud in which it forms, that moderates its response to NRS. The validity of these concepts was further tested by running simulations of the branching of a phylogenetically-distanced prostrate perennial monocotyledonous species, *Tradescantia fluminensis*. For both species the model reasonably accounted for the observed pattern of branching. The outgrowth potential of buds plays an important role in limiting the number of hierarchies of branching that can develop on a plant. In conclusion, for both species, the model accounted for the major factors involved in the correlative regulation of branching and is possibly also pertinent for all prostrate clonal species.

## Introduction

**Fig. 1.**
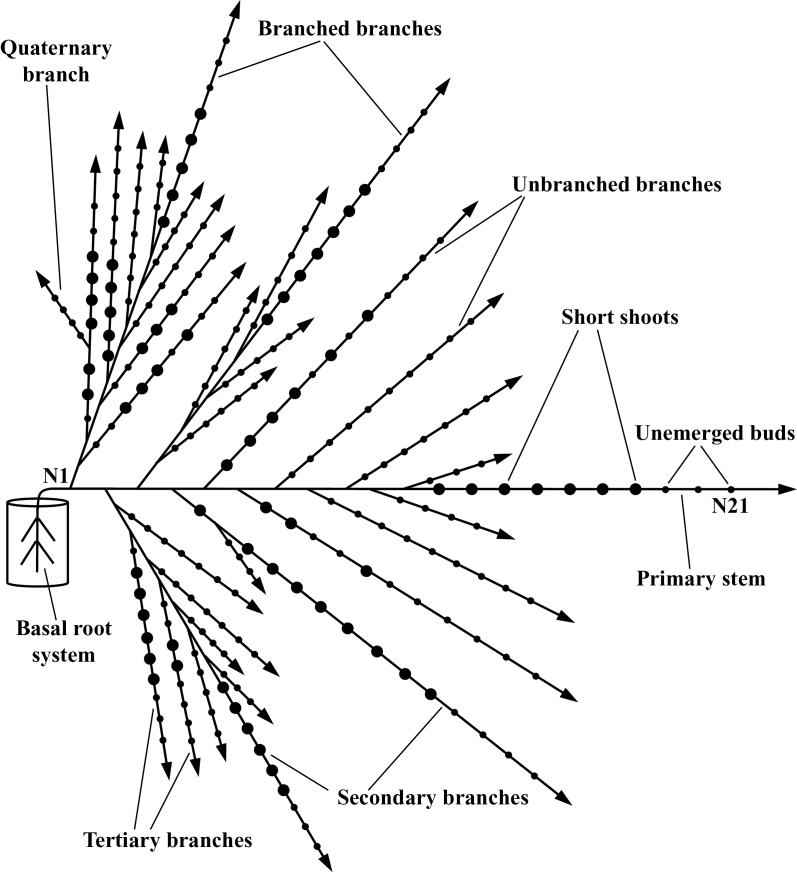
A stylized diagram of mean shoot system morphology of the four greenhouse-grown *Trifolium repens* plants showing branching hierarchy and the four categories of axillary bud development (amended from Thomas and Hay, 2008a and reproduced by kind permission of Oxford University Press): branched branches, unbranched branches, short shoots (large dots), and unemerged axillary buds (small dots). Emerged nodes along the primary stem are numbered acropetally from N1 at the base up to the youngest at N21.

A pattern of branching commonly observed on actively growing shoots of a wide range of plants is one in which early-formed nodes bear strong second order branches that are often themselves further branched to give several higher orders of branching. With increasing distance from the base of the shoot, however, the branching vigour declines rapidly until, ultimately, the latest formed nodes remain unbranched. Such a pattern of decline is particularly apparent in prostrate clonal herbs in which the number of branching orders is restricted to three or four as shown in Fig. 1, but even in the largest woody plants, under the most favourable growing conditions, the number of branching orders rarely exceeds seven (Hallé *et al.*, 1978; Barthélémy and Caraglio, 2007). In this paper, a predictive model is presented, based on our current knowledge of the physiological control of branching in perennial prostrate clonal herbs, that can go some way towards providing a basis for understanding the generality of this phenomenon.

Classical studies into the regulation of branching have used mainly erect annual species grown from seed (such as *Pisum, Petunia*, and *Arabidopsis*). These have focused predominantly on the inhibitory role of apical dominance on axillary bud outgrowth and the parts played by auxin and, more recently, strigolactone therein (Brewer *et al.*, 2009, 2013; Hayward *et al.*, 2009; Prusinkiewicz *et al.*, 2009; Crawford *et al.*, 2010; Liang *et al.*, 2010; Xie *et al.*, 2010; Domagalska and Leyser, 2011; Shinohara *et al.*, 2013). In such species, axillary bud outgrowth is also influenced by the developmental changes that occur as the plants progress towards flowering from an initial vegetative seedling stage (Napoli *et al.*, 1999).

In contrast to the species utilized in the above studies, prostrate perennial clonal herbs can be grown vegetatively from basally rooted cuttings in which a primary stem grows away from a basal root system without progressing towards flowering. Under these circumstances, following an initial phase in which axillary buds grow out vigorously into branches, a very predictable restriction in the branching pattern develops in all ten species that have been examined (Lötscher and Nösberger, 1996; Thomas* et al.*,2002, 2003a; Thomas and Hay, 2004, 2008*b*, 2010), as shown for *Trifolium repens* L. (white clover) in Fig. 1. The usefulness of prostrate clonal herbs for understanding the relationship between roots and axillary bud outgrowth comes from the widespread ability of their nodes to form one or more roots under conditions of high humidity. Controlled stimulation of the formation of a single root in an unbranched region of a stem, such as node 14 in Fig. 1, leads the axillary bud at that node to grow out into an elongated branch. The pattern of branching decline at successively formed unrooted nodes then repeats precisely that occurring in response to the basal root system (Thomas *et al.*, 2002). Using *T. repens* as an exemplar for this group, our work has shown that this stimulatory influence of roots is the dominant factor involved in the regulation of axillary bud outgrowth into branches (Thomas *et al.*, 2002, 2003*a*, *b*; Thomas and Hay, 2007, 2008*a*, 2009). In the erect-stemmed model plant systems described above, however, it has clearly been demonstrated that there is a network of shoot and root feedback and interacting signals that collectively operate to regulate branching (Beveridge, 2006; Aguilar-Martínez *et al.*, 2007; Simons *et al.*, 2007; Ongaro and Leyser, 2008; Ferguson and Beveridge, 2009; Shimizu-Sato *et al.*, 2009; Dun *et al.*, 2009*a*, *b*; Leyser, 2009; Domagalska and Leyser, 2011). In view of this, the stimulatory influence transported from roots in the prostrate-stemmed plants of the present study may well be the net result of both stimulatory and inhibitory influences and is referred to henceforth, for convenience, as the net root stimulus (NRS).

Evidence indicating that the NRS is acropetally transported from roots along shoot stems includes: (i) the consistent absence of a response of axillary buds proximal to a rooted node in contrast to the marked response of distally located ones (Thomas *et al.*, 2002; Thomas and Hay, 2007), (ii) the more pronounced outgrowth of distal buds that have direct vascular connections to the nodal root (Sackville Hamilton and Hay, 1998; Thomas *et al.*, 2002; Thomas and Hay, 2007), and (iii) the immediate cessation of further stimulation of bud outgrowth upon the excision of the local nodal root supplying the stimulus (Thomas and Hay, 2007). The close relationship between duration of exposure to the influence of a nodal root and the rate of axillary bud outgrowth led to the development of the concept of axillary bud activation level. A bud’s activation by the NRS is cumulative and involves the establishment of an activation level within its stem apical meristem (SAM) that is subsequently autonomously regulated to maintain a steady state of SAM functioning for up to at least six weeks even when the supply of NRS from local roots is removed (Thomas and Hay, 2007, 2010). This provides a mechanism allowing a strongly activated meristem, such as the apical bud on a main stem, to continue its growth at a relatively low level of NRS availability while any axillary buds newly emerging from it do so into an environment of low availability and thus remain weakly activated. As a consequence, buds along the same stem, dependent on the same basal root system, can have different activation levels and therefore grow at different rates independently of each other (Thomas and Hay, 2007).

In addition to the level of net stimulus an axillary bud receives, a second factor influencing its rate of outgrowth is its sensitivity to stimuli (Husain and Linck, 1966; Gould *et al.*, 1987; Napoli *et al.*, 1999). This intrinsic ability to respond to the NRS is referred to as ‘outgrowth potential’ (Thomas and Hay, 2009). In *Pisum* (Gould *et al.*, 1987), *Nicotiana* (McDaniel and Hsu, 1976), *Arabidopsis* (Grbic and Bleecker, 2000), and *Arctostaphylos uva-ursi* (Salemaa and Sievänen, 2002) outgrowth potential was related to the developmental stage and physiological activity of the shoot terminal meristem from which the buds were derived. Thomas and Hay (2009) found, in *T. repens*, under standard conditions of NRS supply, the growth rates of parent apical buds directly influenced the rate of outgrowth of the axillary buds that formed within them. In *T. repens* the initial growth rate of an axillary bud is correlated closely to that of the parent apical bud in which it is developing such that, when it emerges, it is producing leaf primordia at the same rate as its parent (Thomas, 1962). This relationship with parent bud activity defines its outgrowth potential.

The purpose of the present report is firstly to test whether the incorporation of outgrowth potential into a previous model, based solely on NRS supply (Thomas and Hay, 2008*a*), improves the fit of model output with observed data. Although the concepts underpinning the model were developed using *T. repens,* its validity for prostrate clonal herbs in general has also been tested by examining its ability to simulate bud outgrowth in *Tradescantia fluminensis* Vell. (wandering jew), a more highly branched, monocotyledonous, species previously found to tolerate experimental manipulations (Thomas and Hay, 2009, 2010).

## Materials and methods

### Definitions

#### Bud activation level (AL):

the intrinsic self-regulatory ability an apical or axillary bud has to grow as assessed by the rate of leaf emergence from the bud relative to that of the bud nearest the root system.

#### Net root stimulus (NRS):

the stimulatory signal that is the resultant of root-supplied stimulatory and inhibitory signals transported acropetally from roots.

#### NRS availability:

the strength of the NRS signal in the shoot system distal to the nearest root at a particular point in time, relative to that available from that root system at the time of its formation.

#### Outgrowth potential (OP):

the potential an axillary bud has to grow in response to a given supply of NRS, relative to that of the axillary bud at the nearest rooted node proximal to it.

#### Threshold activation level:

the critical activation level required in an axillary bud for it to (i) start producing emerged leaves or (ii) induce outgrowth of a branch with three or more emerged leaves.

### Models

In the preliminary model proposed by Thomas and Hay (2008*a*) bud activation was based solely on the pattern of distribution of NRS to each bud. As reported, this model, while satisfactorily predicting secondary branch outgrowth along the primary stem, over-predicted the extent of higher order branching on these secondary branches. It is subsequently referred to as the minus outgrowth potential (–OP) model (see equation A2 below). Following the finding (Thomas and Hay, 2009) that the outgrowth potential of an axillary bud influenced its response to NRS, the –OP model has been modified to include the effect of OP on bud outgrowth. This model is subsequently referred to as the +OP model (see equation A5 below).

The assumptions underpinning both models, and the morphological categorization of bud outgrowth in *T. repens,* are summarized in Table 1 and Fig. 1, respectively. In general terms, the models calculate the activation level of each axillary bud within the shoot system and then use this value to select and apportion to the bud an appropriate outgrowth response from the range of possible responses. These responses of axillary buds, when coupled with their location within the shoot system, are then used to describe the branching phenotype of the shoot system. A more detailed description of the models follows.

**Table 1.** Experimentally established principles relating to the activation of axillary buds by a root-supplied stimulatory signal (NRS) which are incorporated as assumptions underpinning model development

	Principle	References
(1)	Axillary buds are activated by an acropetally transported, root-supplied, net stimulatory signal (NRS)	Thomas *et al.*, 2002, 2003a, *b*
(2)	Availability of NRS to axillary buds upon emergence from their parent apical bud decreases as their phytomeric (nodal) distance from the nearest source root increases	Thomas and Hay, 2008*a*
(3)	Availability of NRS at any one time is uniform throughout the shoot system distal to a nodal root	Thomas and Hay, 2008*a*
(4)	Leaf emergence from axillary buds is directly related to bud activation by NRS whereas stem elongation of buds is indirectly related	Thomas and Hay, 2009
(5)	Each axillary bud acquires an outgrowth potential directly related to the growth rate (activation level) of its parent apical bud	Thomas and Hay, 2009
(6)	The outgrowth potential of an axillary bud modifies its response to the supply of NRS it receives	Thomas and Hay, 2009
(7)	The outgrowth potential of an axillary bud and the supply of NRS to the bud following its emergence from its parent apical bud each independently influence the activation of the axillary bud	Thomas and Hay, 2009
(8)	The activation level attained by an axillary bud is subsequently retained (for at least a 6-week period) even in the face of lowered NRS supply	Thomas and Hay, 2007
(9)	The activation level of each axillary bud within a shoot system is independently determined and maintained	Thomas and Hay, 2007, 2008*a*, 2009
(10)	The minimum threshold level of axillary bud activation required to induce short shoot formation is less than that required to initiate branch formation	Thomas *et al.*, 2002; Thomas and Hay, 2007

The model is primarily based on the geometric decline in availability of NRS throughout the shoot system distal to a basal root system and the influence this has in activating the outgrowth of axillary buds. Firstly there is a direct effect of NRS availability on the activation level (AL) of the axillary bud at the time it emerges from its parent apical bud and, because the distribution of NRS is uniform throughout the plant [Table 1, (3)], at any point in time every newly emerged bud is exposed to the same NRS availability. Then, secondly, the bud response to NRS availability is modified by an indirect (historical) influence of NRS in setting the outgrowth potential (OP) of the axillary bud via its previous influence on the activation level of its parent apical bud (see schematic representation in Discussion). This gives rise to the basic relationship driving the model for calculation of axillary bud activation level:


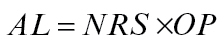
(A1)

Both NRS and OP can be expressed in relation to the supply of NRS as described below. NRS is a relative measure, scaled from unity (1) at the oldest node to 0 at the youngest. The OP, as defined below, is also a relative measure but is expressed as a percentage which means the resultant AL is also scaled from 100 to 0.

#### NRS availability:

For the non-rooted shoot system developing beyond the basal root system, it is assumed, based on Thomas and Hay (2008*a*), that the level of NRS available at the sites of successively emerging buds decays in the form of a geometric series, with a common ratio *R* (*R*=0.9 in Thomas and Hay, 2008*a*). *R* is set by the number of nodes present on the primary stem such that the AL decays to <0.5 at the youngest emerged node and so only represents the size of the plant.

The NRS available at a node (or axillary bud) at any position in the shoot system, at the time of its emergence from its apical bud, can be determined by its distance (measured as the number of nodes) from the basal root system, incorporating an adjustment (*L*) for the delay associated with the development of a branch relative to parent stem growth (also measured as a number of nodes). This arises because buds are absent from the axils of the youngest few leaf primordia within a vegetative apical bud (Thomas, 1987). Dissection of apical buds showed that the delay was similar in both *T. repens* and the *Tradescantia* plants of this study. As a result, in both species, a branch forming from a fully activated bud has ~3 nodes fewer than occur on the stem bearing it distal to its point of attachment. This means that the NRS at a particular node in a branch of *n* ^th^ branching order is


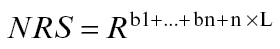
(A2)

where (*b1+...+bn*) is the distance (number of nodes) between the node and the rooted node, with *b1, b2, b3,...bn* referring to the number of nodes the NRS has to travel on the branches of branching orders 1, 2, 3,...*n*, respectively and *L* is the delay associated with branch development (number of nodes delayed).

#### Outgrowth potential (OP):

The OP of an axillary bud is positively correlated with the growth rate (activation level) of the stem apical bud from which it is derived (Thomas and Hay, 2009). As the apical bud of a stem retains its initial maximal activation level (Thomas and Hay, 2007), all the axillary buds formed by that bud have the same OP, although the OP of axillary buds on different stems will differ because each apical bud has its own activation level (Thomas and Hay, 2007, 2009). That is, axillary bud OP is in proportion to the AL of its parent apical bud (axillary bud OP=*k*×parent bud AL, where *k* is a constant); however, as the relationship between axillary and apical bud growth rate is linear (Thomas and Hay, 2009), the parental bud AL is used to quantify the axillary bud OP.


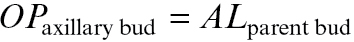
(A3)

By definition, the apical bud on the primary stem and the axillary bud at the node nearest to the basal root are fully activated and hence have an activation level of 100 units so all axillary buds on these stems will have an outgrowth potential (OP) of 100. However, the activation levels of the apical buds on the secondary branches along the primary stem will decrease with distance from the basal root system according to the decrease in NRS (*R* ^b1^). Thus the OP of an axillary bud formed on the secondary branch arising at node *b1* on the primary stem will be:


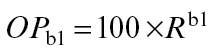


The OP for the axillary buds on the tertiary branch forming at node *b2* on the secondary branch at *b1* will be a function of the activation level at that node


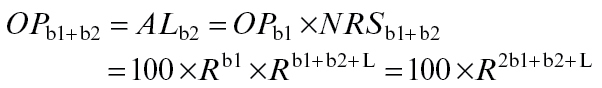


In this way OP can be ascribed to each axillary bud in the shoot system according to the position of the bud as follows:


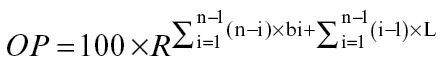
(A4)

where *n* is the order of the branch at which the bud is located; *i* enumerates the order of branch from the first to the *(n–1)* ^th^ *, b1…bn* and *L* are the same as in equation (A2); 
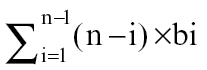
 is the cumulative distance (the number of nodes) NRS has to travel to reach the parent apical bud at the branch of (*n*–*1)* ^th^ order; and 
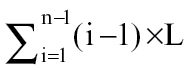
 is the cumulative delay in branch development arising from each of the *n–1* branching orders for the bud on the *n* ^th^ branching order stem.

*Axillary bud activation level (AL)*: As per the definition in equation (A1), the AL of an axillary bud on a *n* ^th^ order branch is the product of its NRS and parent bud OP:


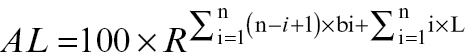
(A5)

where all the variables are the same as in equation (A4)

**Fig. 2.**
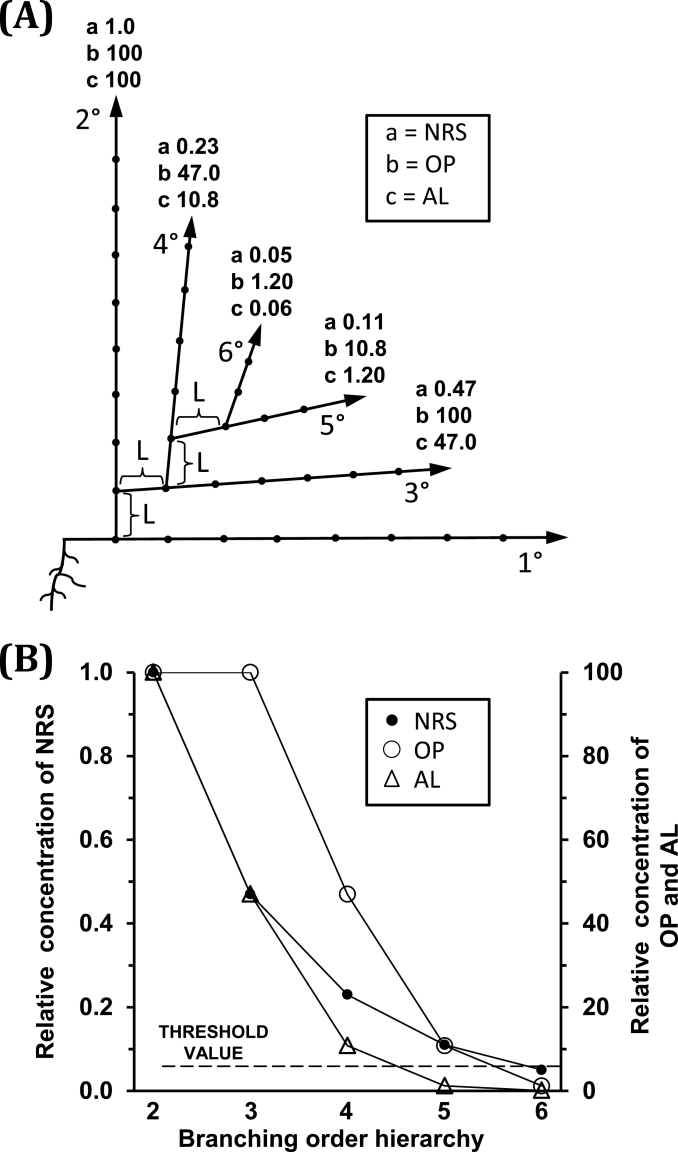
A diagrammatic representation (A) of the relative values for *Trifolium repens* of outgrowth potential (OP), net root stimulus (NRS), and activation level (AL) of the axillary bud at node position 1 on the primary stem (branching order 1) and for the axillary buds at the first node on each branch of the subsequent hierarchies of branching that could occur on a branch system developing at that node position (calculated where *R*=0.78 and *L* (delay in branch development) =2) with *L* indicated at each level of branching hierarchy; and (B) a plot of the values of OP, NRS, and AL showing the effect of OP in accelerating the reduction in AL as branching hierarchy increases so that AL is rapidly reduced below the threshold value of 6 (broken line) for branch outgrowth.

The relationships among NRS, OP, and AL and their decline across branching orders in *T. repens* are diagrammatically presented in Fig. 2. The effect of *L,* the delay between branching hierarchies before branch development commences, is shown in Fig. 2A along with the values of each of the parameters for the first node on the branch of each successive hierarchy of branching. The rapid decline in AL with increasing branching order and its relationship to the threshold value for branch outgrowth is shown in Fig. 2B.

#### Threshold activation levels:

The activation levels (AL) of axillary buds on the primary stem at the node with the youngest short shoot (node 18 in Fig. 1) and at the node at the distal end of the zone of branches (node 11 in Fig. 1) determine, respectively, the activation thresholds for short shoot and branch outgrowth. A consequence of these two activation thresholds is that axillary buds can then be allocated to one of three categories of morphological activity: (i) at activation levels below both thresholds buds remain unemerged, (ii) at AL at and above the threshold for short shoot formation, but below that for branch outgrowth, axillary buds produce 1 to <3 leaves to form short shoots, and (iii) at AL at and above the branch outgrowth threshold axillary buds grow out into branches bearing three or more leaves. For a given set of experimental conditions the threshold values are considered an intrinsic characteristic of a species or genotype.

#### Shoot system morphology:

With increasing levels of bud activation, there are four possible categories of axillary bud development: unemerged buds (buds with <1 emerged nodes), short shoots (buds with 1 but <3 emerged nodes), unbranched branches (buds with ≥3 emerged nodes), and branched branches (Fig. 1). Calculation of the activation level of each axillary bud thereby allows prediction of the branching morphology of the whole shoot system.

#### Model parameterization:

The common ratio (*R*) for geometric decline of NRS is defined such that the activation level (AL) of the youngest emerged node just proximal to the apical bud on the primary stem has a value of 0.5.

Assuming the primary stem has *b* nodes, then we have 100*R* ^b^=0.50, which solved gives


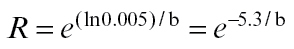
(A6)

### Parameterization for *T. repens*

As the *T. repens* grown in experimental pots had 21 emerged nodes on the primary stem (Fig. 1), *R*=0.78. The observed delay in development between each branching order (*L*) is 2.

On the primary stem, the first 11 nodes have branches and an AL>6, and nodes 12–18 have short shoots with 1–3 emerged leaves with 6>AL>1.3. The threshold AL values of 6 and 1.3 are applied to each bud in the shoot system to determine its category of bud development.

### Parameterization for *Tradescantia*

As the *Tradescantia* grown in the experimental pots also had 21 emerged nodes on the primary stem, it also had *R*=0.78. The observed delay in development between each branching order (*L*) is 2.

On the primary stem of the experimental plants, the first 16 nodes had branches with AL>1.6, and at nodes 17–18 there were short shoots with AL in the range 1.6>AL>1. The threshold AL values (1.6 and 1.0) are applied to each bud in the shoot system so as to predict its category of bud development.

Because the oldest four branches on the *Tradescantia* plants were excised when the first eight nodes had emerged on the primary stem, each of the remaining four buds initially would have received the same high level of NRS (*R*=1). To accommodate this growth artefact, the decay in NRS availability was adjusted to commence at node 5.

### Observed branching phenotypes

#### Trifolium repens L.:

Four plants of a single genotype of *T. repens* were grown from cuttings taken from a stock plant under previously described conditions (Thomas *et al.*, 2002). Each cutting of two nodes plus the apical bud was planted into a 5.0 l pot of standard potting mix. They were planted such that they subsequently grew out over the edge of the pot to form a stem which was non-rooted except for the root systems forming at the two basal nodes. After 55 d growth, when the stem had produced 21 non-rooted nodes, the branching phenotype was recorded by counting the number of emerged nodes on each branch on the non-rooted shoot system.

#### Tradescantia fluminensis Vell.:

Three plants of *Tradescantia* were similarly grown from cuttings of a single genotype. Each cutting of two nodes plus the apical bud was planted into a 5.0 l pot of standard potting mix and then grown out over the edge of the pot to form a non-rooted shoot system distal to the two basal rooted nodes. After the emergence of eight nodes distal to the basal roots, leaves and their subtended axillary buds were excised from the oldest four. The primary stem on each was then allowed to grow for a further 17 nodes. The branching phenotype of these 21-node plants was then characterized by counting the number of nodes on each branch of the non-rooted shoot system.

### Comparison of models with observed branching

Output generated by each of the models was compared with the branching pattern observed in the greenhouse-grown plants. As each model was parameterized by the outgrowth of buds along the primary stem, the test of its predictive ability had to be based on comparisons of the extent of higher order branching. Thus the predictions by the models of the numbers of branches (or, for Fig. 4B, number of short shoots) in the various hierarchies of branching (3^rd^, 4^th^, and 5^th^ order) on secondary branches at each node position along the primary stem were compared with the numbers observed on the greenhouse-grown plants. The Kolmogorov–Smirnov test (Siegel, 1956; Marsaglia *et al.*, 2003), which assesses the probability of two samples coming from the same distribution, was used to compare the observed versus predicted values for each branch order. Since the values compared here are discrete in nature a ‘discrete Kolmogorov–Smirnov test’ is utilized (Arnold and Emerson, 2011).

## Results

### Trifolium repens

**Fig. 3.**
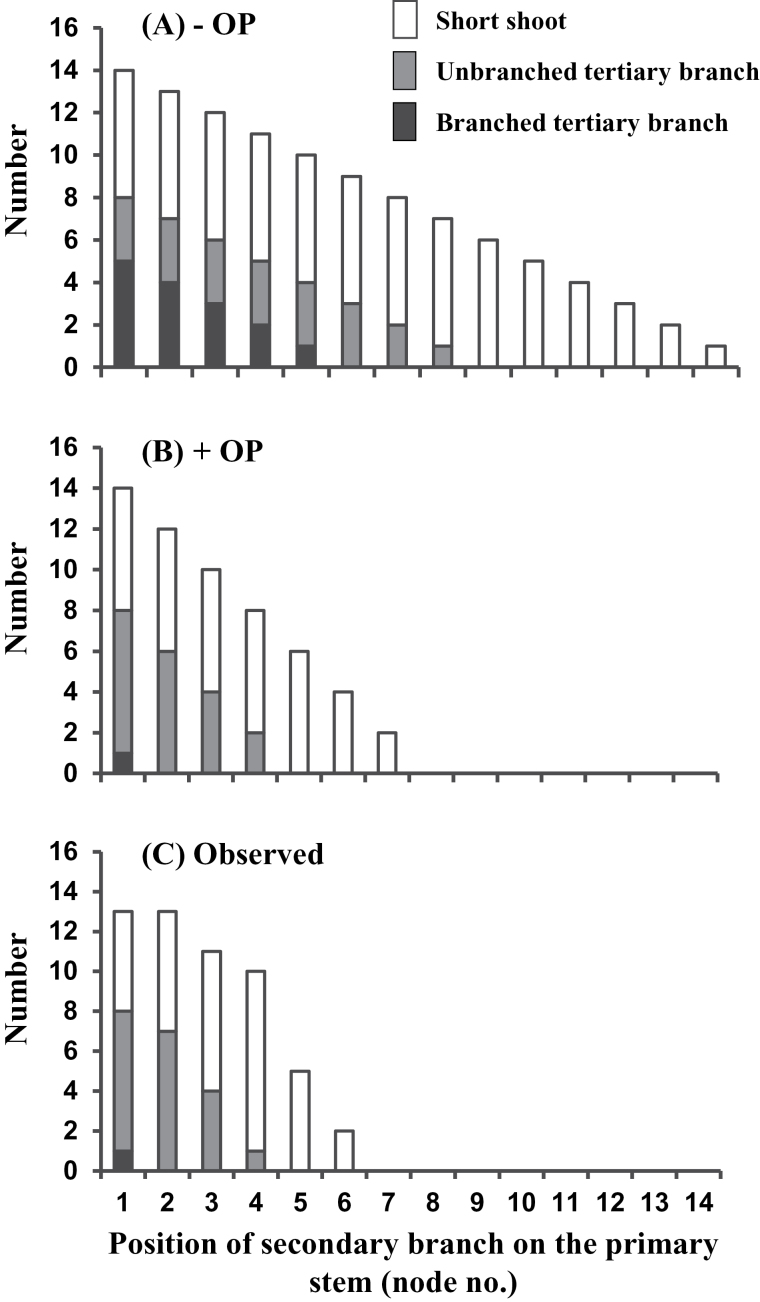
For *Trifolium repens* the number of nodes bearing branched 3^rd^ order branches, unbranched 3^rd^ order branches or 3^rd^ order short shoots on the secondary branch at each node position along the primary stem as predicted by (A) the –OP model, (B) the +OP model and (C) as observed on greenhouse-grown plants (*n*=4).

Characteristics of the 3^rd^ order outgrowth from the axillary buds on the secondary branches at each node position along the primary stem predicted by the –OP and +OP models differ greatly (Fig. 3A versus B). Whereas the +OP and the observed values (Fig. 3B versus C) are quite similar, with both showing short shoot outgrowth only up to nodes 6 or 7 on the primary stem, the –OP model predicted that short shoot outgrowth would extend as far as node 14.

**Fig. 4.**
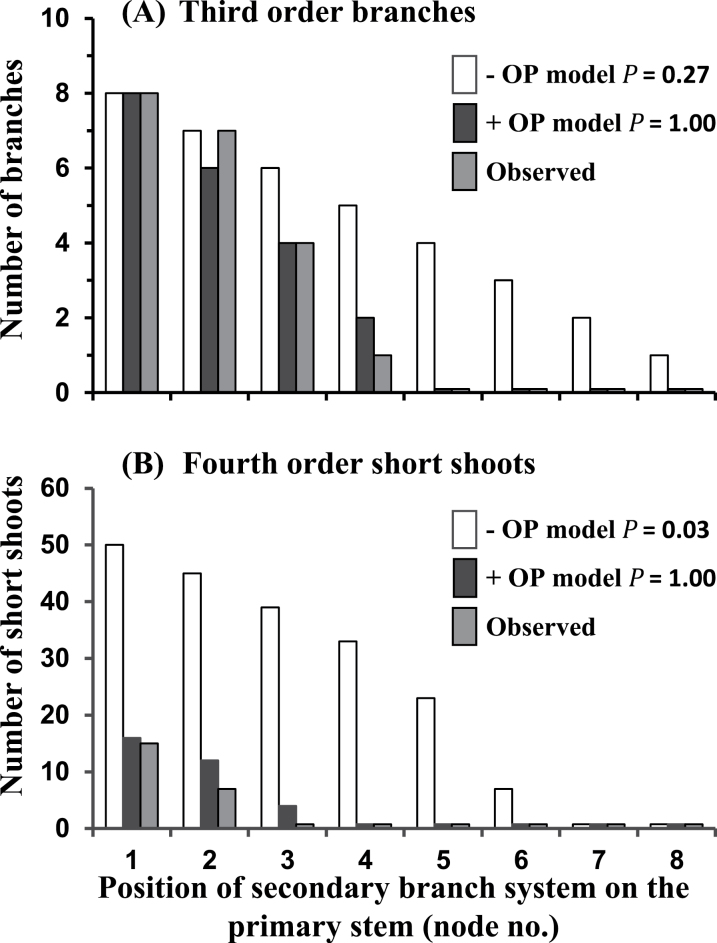
For *Trifolium repens* (A) the numbers of 3^rd^ order branches (unbranched plus branched branches but excluding short shoots) and (B) the total numbers of 4^th^ order short shoots on the branch systems at each node position along the primary stem as predicted by the –OP model, the +OP model or as observed on greenhouse-grown plants (*n*=4). For both the –OP and +OP models the probability (*P*) (Kolmogorov–Smirnov test) that the predicted and observed values are from the same distribution is given.

Numbers of 3^rd^ order branches predicted by the –OP model also tended to be greater than the observed values at the distal-most nodes on the primary stem (Fig. 4A), although not significantly so as indicated by the conservative Kolmogorov–Smirnov test, whereas the number predicted by the +OP model is markedly lower and the same as on the observed plants. Similarly, the –OP model incorrectly predicts the presence of a total of 15 branched tertiary branches at node positions 1–5 on the primary stem (Fig. 3A) while, again, the +OP model matches the observed value by accurately predicting the presence of just a single such branch located at node 1 (Fig. 3B, C).

In the almost complete absence of 4^th^ order branches, the number of 4^th^ order short shoots was analysed to assess the predictability of the models at the quaternary level (Fig. 4B). As at other levels, the –OP model grossly over-predicted both the number and the extent of their occurrence along the primary stem but the +OP model closely tracked the observed values.

### Tradescantia fluminensis

**Fig. 5.**
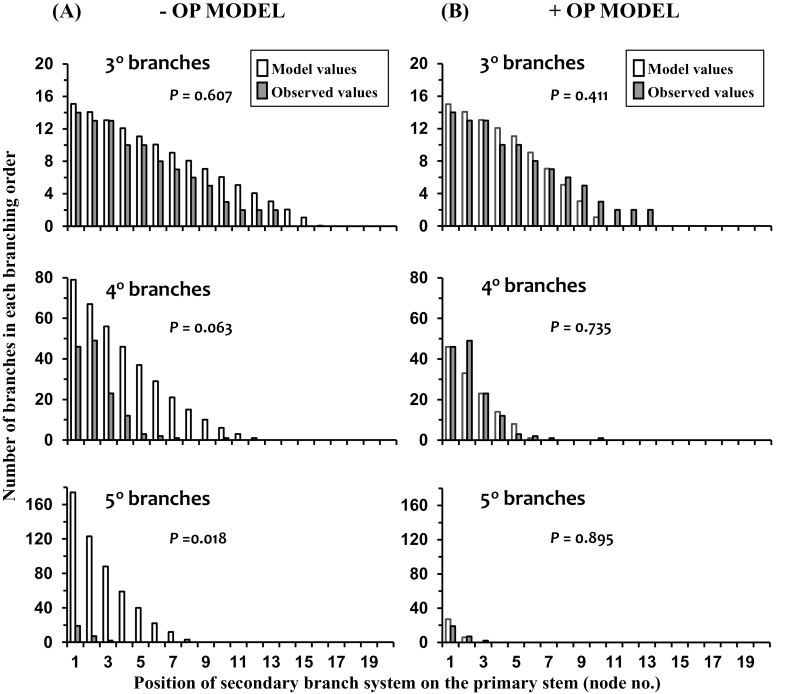
For *Tradescantia fluminensis* the predictions by (A) the –OP model and (B) the +OP model, compared with the observed values (*n*=3), for the total numbers of 3^rd^, 4^th^, and 5^th^ order branches on secondary branch systems at each node position along the primary stem. For each branching order, the probability (*P*) (Kolmogorov–Smirnov test) that the predicted and observed values are from the same distribution is given.

Numbers of 3^rd^ order branches predicted by the –OP model (Fig. 5A) were not significantly greater than observed values. However, relative to the observed values, the predicted numbers of 4^th^ (*P*=0.063) and 5^th^ (*P*=0.018) order branches at each node position along the primary stem, respectively tended to be, or were significantly, overestimated.

The estimates of branch numbers predicted by the +OP model (Fig. 5B) for each of the three branching hierarchy levels (3^rd^, 4^th^, and 5^th^ branch orders) closely matched, and did not significantly differ from, the observed values.

## Discussion

The similarity of the branching phenotype predicted by the +OP model and that observed on experimental plants for both *T. repens* (Figs 3, 4) and *Tradescantia* (Fig. 5B) implies that this model accounts for the major factors determining outgrowth of axillary buds under the experimental conditions described (Thomas *et al.*, 2002). The other possibility, that the accurate predictions by the +OP model result from compensating errors, is an unlikely scenario because this model is a further development of the earlier –OP model (Thomas and Hay, 2008*a*). The –OP model, based solely on intra-plant distribution of NRS, was identified as over-predicting branching on the higher hierarchies of plant branching (Thomas and Hay, 2008*a*) and was again found, in this study, to do so (Figs 3, 4, 5A). The highly predictive nature of the +OP model, based on the distribution pattern of a positive signal from roots along with modification of its effectiveness by a bud’s outgrowth potential, strongly supports the suggestion that these two parameters can account, in the main, for the observed branching pattern in both these species.

The model is primarily driven by the geometric decline in the decay of NRS (*R*) with nodal distance (or plastochron interval) from the basal root system. The absolute value of *R* merely reflects plant size (number of nodes on the primary stem) and does not affect model output. Model output is sensitive to *L,* the delay (assessed as number of plastochrons) associated with bud outgrowth relative to parent stem growth, as the smaller the delay the more highly branched is the output phenotype. Model output is very sensitive to the species-specific settings of threshold levels for branch outgrowth and bud emergence, these being set by calibration from the primary stem. For instance, *T. repens* and *Tradescantia* both have a delay (*L*) of 2 and a NSR decay rate (*R*) of 0.78, but different threshold values (6 and 1.3 versus 1.6 and 1.0, respectively for branch outgrowth and bud emergence) mean the species have very contrasting branching patterns (Figs 4, 5). This sensitivity to threshold values confers the model with potential to cope with great interspecific variation in branching patterns ranging from the sparsely branched guerrilla species such as *Calystegia silvatica* and through to the much branched phalanx species such as *Tradescantia* (Thomas and Hay, 2010).

For both *T. repens* and *Tradescantia,* comparison of the predictive values from the –OP and +OP models (Figs 4 and Fig. 5A versus 5B, respectively) demonstrates the very significant effect the outgrowth potential (OP) of an axillary bud has in determining the branching phenotype of these species. A decline in OP is clearly an important factor reducing bud outgrowth as nodal distance from roots increases and as branching hierarchy increases. For *T. repens*, Fig. 2 demonstrates the accelerated decrease in AL that occurs with increased branching hierarchy as a result of decreased OP reducing the effectiveness of NRS. Indeed, comparison of the –OP and +OP models for *Tradescantia* indicates that the restriction of higher order branching, to a maximum of 6^th^ order, so as to occur only at those secondary branches closest to the root system, requires the involvement of an OP influence. The restriction of plant branching order to a maximum of seven is common throughout the plant kingdom, except for some small-leaved species (Hallé *et al.*, 1978; Barthélémy and Caraglio, 2007), and the influence of bud outgrowth potential may be a significant factor in the regulation of this limitation.

This study suggests, therefore, that, in *T. repens* and *Tradescantia*, the observed pattern of declining branching is the result of double jeopardy, being dependent almost entirely on the decline in NRS availability as the shoot system grows away from the basal root system. This decline has a dual negative effect on axillary bud outgrowth, firstly by directly influencing a bud’s activation level and, secondly, by indirectly determining its OP by affecting the activation level of its parent apical bud. The central role of the net root stimulus (NRS) can thus be summarized as below:

**Figure:**
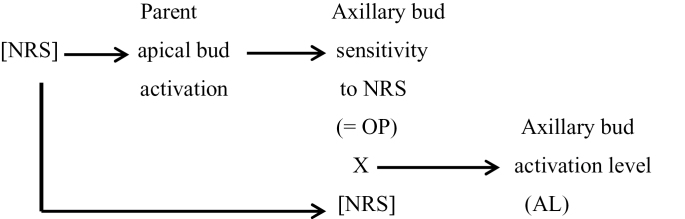


Ferguson and Beveridge (2009) suggest the regulation of bud outgrowth in pea is via the three independent but interacting inhibitory processes of apical dominance (auxin), the strigolactone pathway, and correlative inhibition. In *T. repens*, however, apical dominance effects are small, being minimal at buds 15 or more from a root (Thomas *et al.*, 2003*b*); root signals are stimulatory rather than inhibitory suggesting the strigolactone pathway either plays a limited role or, possibly more likely, its effect is captured within the net stimulatory effect (NRS); and correlative inhibition induced by existing branches has the dominant effect. This correlative inhibition does not appear to be mediated by a phloem-mobile inhibitory signal or auxin feedback from branches but is consistent with branches moderating the distribution of NRS (Thomas and Hay, 2011). Hence, in *T. repens*, the relative significance of the three known inhibitory processes regulating branching differs from the effects they have in pea (Ferguson and Beveridge, 2009). Thomas and Hay (2004) suggested that, as the positive association between nodal root and branch development enhances resource acquisition (Hutchings and Mogie, 1990), the evolution of physiological mechanisms linking their mutual development would be favoured. Hence it is, perhaps, not surprising that there are big differences in the regulation of axillary bud outgrowth between erect annuals and the prostrate perennial, clonal herbs (Thomas and Hay, 2009).

*T. repens* is considered to sit in the middle of the ‘phalanx–guerrilla continuum’ (Lovatt-Doust and Lovatt-Doust, 1982) for prostrate clonal herbs, and is therefore studied as an exemplar of the group (Thomas and Hay, 2008*b*, 2010), whereas the monocotyledonous *Tradescantia* has a bunched growth habit characteristic of species at the phalanx end of the continuum (Thomas and Hay, 2010). *Calystegia silvatica, Glechoma hederacea*, and *Lamiastrum galeobdolon* are all at the guerrilla end of the continuum and have been examined to test the hypothesis that bud outgrowth throughout the continuum is similarly regulated. In all cases, branching was stimulated by nearby nodal roots (Thomas and Hay, 2004, 2008*b*) with this stimulation being cumulative (Thomas and Hay, 2010). Hence, as the model was proved pertinent for the phalanx and middle portions of the continuum, our evidence, to date, is consistent with the major elements of the model having general applicability over the whole range of the phalanx–guerrilla continuum for prostrate clonal herbs.
